# Engineering alternative isobutanol production platforms

**DOI:** 10.1186/s13568-015-0119-2

**Published:** 2015-06-04

**Authors:** Carmen Felpeto-Santero, Antonia Rojas, Marta Tortajada, Beatriz Galán, Daniel Ramón, José L García

**Affiliations:** Department of Environmental Biology, Centro de Investigaciones Biológicas, CSIC, Madrid, Spain; Biopolis S.L., Parc Científic Universitat de Valencia, Paterna, Spain

**Keywords:** Isobutanol, Sucrose, Synthetic operon, *E. coli*, *S. blattae*

## Abstract

A synthetic inducible operon (IbPSO) expressing *alsS*, *ilvC, ilvD* and *kivD* genes encoding a pathway capable to transform pyruvate into 2-isobutyraldehyde has been designed and two recombinant plasmids named pIZIbPSO and p424IbPSO were constructed. The IbPSO containing plasmids can generate in a single transformation event new recombinant isobutanol producer strains and are useful for testing as suitable hosts wild type bacteria in different culture media. In this way we found that *Shimwellia blattae* (p424IbPSO) was able to produce in flasks up to 6 g l^−1^ of isobutanol using glucose as carbon source. Moreover, for the first time, we have demonstrated that isobutanol can be produced from sucrose using *Escherichia coli* W (ATCC9367) transformed with pIZIbPSO. These robust recombinant strains were also able to produce isobutanol from a raw carbon source like hydrolysed lignocellulosic biomass.

## Introduction

The growing concerns on climate change have increased the interest in producing new fuels from renewable sources. Although ethanol provides the first model for biofuel commercialization, different efforts have been made to develop new processes for producing novel advanced biofuels such as C3–C5 alcohols containing higher energy density than ethanol. These alcohols are compatible with the current fuel infrastructures since they are less hygroscopic than ethanol. Two examples of these alcohols are 1-butanol and isobutanol that can be blended with gasoline and also can be used for gasoline replacement, as they perform well in conventional gasoline engines. Taking advantage of the increasing genomic information and the rapidly evolving techniques and tools of metabolic engineering, considerable progress has been made toward developing recombinant microbial strains for the production of these new fuels.

Depending on their metabolic capabilities, heterotrophic microorganisms produce central metabolites such as pyruvate and acetyl-CoA using different external carbon and energy sources including sugar, glycerol, soluble and insoluble polysaccharides, alginates, proteins and other organic compounds. Biosynthetic pathways for producing higher alcohols elongate and reduce these central metabolites into more electron rich compounds, like higher carbon acyl-CoAs and 2-keto acids. However, only few microorganisms are capable to naturally produce isobutanol in low amounts.

Nowadays it is possible to generate precursors for higher chain alcohol production exploiting the amino acid biosynthesis pathways (Atsumi et al. 2008; Chen et al. 2011; Huo et al. 2011; Kondo et al. 2012; Brat et al. 2012). These pathways produce amino acids through 2-keto acid precursors, which can be used as substrates for the Ehrlich biosynthetic pathway to alcohols that consists of two steps, i.e., a decarboxylation of a 2-keto acid to the corresponding aldehyde by a 2-keto acid decarboxylase, followed by a reduction of the aldehyde by an alcohol dehydrogenase. Using this strategy, a non-natural isobutanol biosynthetic pathway has been created by combining genes from different organisms. In this way isobutanol production has been achieved in several engineered microorganisms (for a summary see Akita et al. 2014) like *Escherichia coli* (Atsumi et al. 2008; McEwen and Atsumi 2011; Akita et al. 2014), *Corynebacterium glutamicum* (Blombach et al. 2011), *Bacillus subtillis* (Li et al. 2011), *Saccharomyces cerevisae* (Chen et al. 2011; Kondo et al. 2012; Lee et al. 2012), *Clostridium cellulolyticum* (Higashide et al. 2011), *Synechococcus elongatus* (Atsumi et al. 2009; Lan and Liao 2013), *Pseudomonas* sp (Lang et al. 2014) and *Ralstonia euthropha* (Lu et al. 2012; Li et al. 2012).

Although the synthesis of isobutanol using the keto acid pathway has opened the possibility for its industrial production, the non-native engineered pathways may interfere with the cell metabolism by competing for essential precursors for growth or maintenance. Therefore, to create new fine-tuned pathways that can be perfectly integrated to be compatible with the host cellular metabolism is highly desirable. In this sense, operons have been naturally created to tightly control many pathways and therefore, it appears that assembling the non-natural pathways into well controlled and designed synthetic operons could render some benefits.

In this work we have explored the possibility of constructing a new chemically synthetic operon to transfer the capacity to produce isobutanol to different bacteria, allowing the possibility to test in a simple way many different host organisms as putative isobutanol producers and to regulate it in an integrated manner. Moreover, this approach provides the proof of concept and the opportunity to easily incorporate novel properties to the process (e.g., the use of different raw materials as carbon sources) depending on the strains used as hosts.

## Materials and methods

### Bacterial strains, plasmids, growth media and transformation

The bacterial strains and plasmids used in this study are listed in Table 1. *E. coli* and *Shimwellia blattae* strains were cultured in solid LB medium at 37°C. Antibiotics were used if indicated at the following concentrations: gentamicin (10 µg ml^−1^), streptomycin (50 µg ml^−1^) and ampicillin (100 µg ml^−1^). *E. coli* and *S. blattae* recombinant strains were cultured in M9 minimal liquid media containing a mixture of trace elements (nitrilotriacetic acid 1.5 mg l^−1^, MgSO_4_·7H_2_O 3 mg l^−1^, ZnSO_4_·7H_2_O 0.18 mg l^−1^, CuSO_4_·5H_2_O 0.01 mg l^−1^, MnSO_4_·2H_2_O 0.5 mg l^−1^, NaCl 1 mg l^−1^, FeSO_4_·7H_2_O 0.1 mg l^−1^, CoSO_4_·7H_2_O 0.18 mg l^−1^, NaSeO_3_·5H_2_O 0.3 mg l^−1^, KAl(SO_4_)_2_·12H_2_O 0.02 mg l^−1^, H_3_BO_3_ 0.01 mg l^−1^, Na_2_MoO·2H_2_O 0.01 mg l^−1^, NiCl_2_·6H_2_O 0.025 mg l^−1^) according to Atsumi et al. (2010). This medium was always supplemented with yeast extract (5 g l^−1^). The sugar carbon sources used in isobutanol production experiments were: glucose (20 g l^−1^), xylose (20 g l^−1^), sucrose (20 g l^−1^), or liquid lignocellulosic hydrolysate (wheat straw partially degraded after treatment with acid and elevated temperatures) being its main components: Glucose (59.16 g l^−1^), xylose (27.2 g l^−1^), arabinose (3.04 g l^−1^), galactose (1.42 g l^−1^), cellobiose (1.04 g l^−1^), manose (0.55 g l^−1^), acetic acid (3.73 g l^−1^), formic acid (0.14 g l^−1^), hydroxymethylfurfural (0.39 g l^−1^), Furfural (0.77 g l^−1^), 4-hydroxybenzoic acid (0.009 g l^−1^), vanillin (0.029 g l^−1^), syringaldehyde (0.016 g l^−1^), cumaric acid (0.11 g l^−1^), and feluric acid (0.15 g l^−1^) (provided by Biopolis S.L.). The hydrolysate was included in the minimal medium at different concentrations as indicated in volume percentages (% vol/vol, i.e., ml of liquid hydrolysate in 100 ml of total culture medium). The hydrolysate cannot be used without dilution as a culture medium since it is toxic for the bacteria tested in this study when used at concentrations higher than 50% (vol/vol). Antibiotics were used for plasmid maintenance at the following concentrations: Gentamicin (10 µg ml^−1^) or streptomycin (50 µg ml^−1^). Cells were cultured overnight in LB (10 ml) in 100 ml flasks at 37°C in an orbital shaker at 250 rpm. Cells were inoculated at 0.05 OD_600nm_ in production media (10 ml) in 100 ml screw flasks at 37°C in an orbital shaker at 250 rpm during 2.5–3 h, until reaching an OD_600nm_ of 0.5. Then, cultures were induced by adding 1 mM IPTG and grown at 30°C in an orbital shaker at 200 rpm during 48 h. This culture process has been performed either in shake flask or in 96-microwell plates. Culture growth was monitored with a Shimadzu UV-260 spectrophotometer in shaking screw flasks. For the cultivation in 96-microwell plates, aliquots (0.2 ml) were distributed in the microwells. The plates were incubated at same temperature for 48 h, with 20 s of heavy orbital shaking every 15 min using a Multiskan Ascent Incubator (Thermo Scientific, Waltham, MA, USA) that monitors optical density at 630 nm every 60 min. Transformation of bacterial strains was performed by electroporation as previously described (Díaz et al. 1994).

**Table 1:** Bacterial strains and plasmids used in this study

Strains	Genotype	References
*Escherichia coli* DH10B	F^−^, *mcrA*, *Δ*(*mrr*-*hsdRMS*-*mcrBC*), *f80ΔlacZDM15* *ΔlacX74*, *deoR*, *recA1*, *endA1*, *araD139*, *Δ*(*ara,leu*)*7697*, *galU*, *galK*, *rpsL*, *nupG*, *λ* ^−^	Invitrogen
*Escherichia coli* W	Type strain. Waksman’s strain	ATCC9637
*Shimwellia blattae*	Type strain	CIP 104942, DSMZ 4481
Plasmids		
pUC57	Ap^R^, pUC-derived cloning vector for *E. coli* bearing *Plac*. *rep* (pMB1)	Genscript
pIZ1016	Gm^R^, broad-host range expression vector bearing *lacIq* and *Ptac. rep* (pBBR1MCS)	Martínez-Perez et al. (2004)
pSEVA424	Sm^R^/Sp^R^, broad-host range expression vector bearing *lacIq* and *Ptrc.rep* (RK2 replicative origin. *oriV*-*trfA*)	Silva-Rocha et al. (2013)
pUC57-IbPSO	Ap^R^, IbPSO into pUC57	This work
pIZIbPSO	Gm^R^, IbPSO into pIZ1016	This work
p424IbPSO	Sm^R^/Sp^R^, IbPSO into pSEVA424	This work

### Construction of the synthetic operon

**Figure 1:**
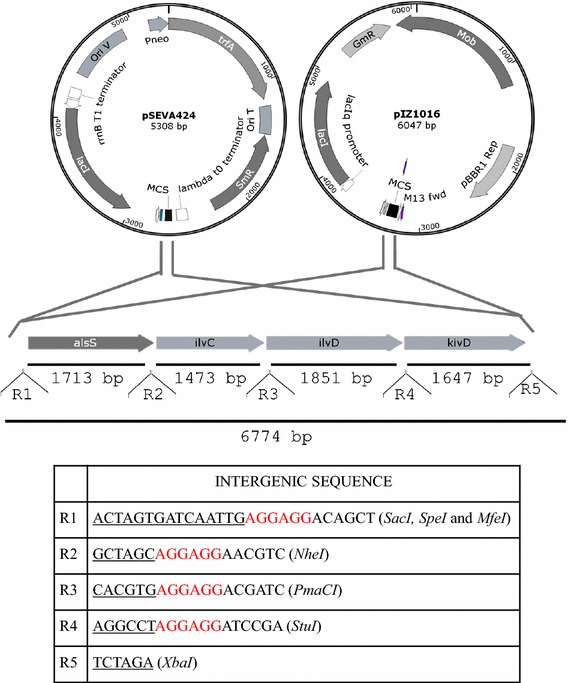
Schematic representation of the genes contained in the IbPSO synthetic operon. The sequences of the intergenic regions (R1–R5) are indicated in the *table*. The sequences of the restriction sites are *underlined* and the corresponding restriction enzymes are *annotated*. The RBS sequences (AGGAGG) are indicated in *red*. The maps of the two broad host range plasmids, pSEVA424 and pIZ1016, used to clone the IbPSO operon are shown.

The synthetic operon (Figure 1) (GenBank accession No. KP739244), named IbPSO, was designed to express the following genes: *alsS* (acetolactate synthase) from *B. subtilis, ilvC* (acetohydroxy acid isomeroreductase) from *E. coli*, *ilvD* (dihydroxy-acid dehydratase) from *E. coli* and *kivD* (2-ketoacid decarboxylase) from *Lactococcus lactis*. To achieve optimal translation of the mRNA a consensus Shine Dalgarno sequence (AGGAGG) was added upstream each gene at 6 bp from the respective start codons. Restriction enzyme sites were added upstream each gene to facilitate different cloning options. Codon usage was adapted to Gram negative bacteria using the Optimizer program (Puigbó et al. 2007, 2008) at Universitat Rovira i Virgili server (http://genomes.urv.es/OPTIMIZER/). IbPSO operon was chemically synthesized at ATG:biosynthetics GmbH and was initially cloned into pUC57 generating the plasmid pUC57-IbPSO. Plasmid pUC57-IbPSO was digested with *Spe*I-*Xba*I or *Sac*I-*Xba*I to release the fragment containing the IbPSO operon that was further subcloned into the pIZ1016 (under the control of the *Ptac* promoter) and pSEVA424 (under the control of *Ptrc* promoter) vectors, respectively (Figure 1).

### HPLC analysis

For quantification of isobutanol 80 µl of culture supernatants were precipitated by the addition of 10 µl of both 0.1 M Ba(OH)_2_ and 0.1 M ZnSO_4_, and after removing the precipitate by centrifugation at 12,000*g* for 5 min, supernatants were analyzed by HPLC using a Gilson HPLC system equipped with a refraction index detector and an Aminex HPX-87H column (300 × 7.8 mm, hydrogen form, 9 µm particle size, 8% cross linkage). The column was eluted isocratically at a flow rate of 0.6 ml min^−1^ of 5 mM H_2_SO_4_. The analysis was performed using Beckman Coulter’s *32 Karat Software* 8.0.

### Isobutanol and norvaline tolerance test

To test isobutanol tolerance bacterial strains were inoculated at an OD_600nm_ of 0.01 in M9 liquid medium supplemented with trace elements as described above, yeast extract (5 g l^−1^), glucose (36 g l^−1^) and isobutanol (0–10 g l^−1^). Growth was determined at 24 h by determining the O.D at 600 nm. Norvaline tolerance was tested according to Smith and Liao (2011).

## Results

### Construction of IbPSO operon

**Figure 2:**
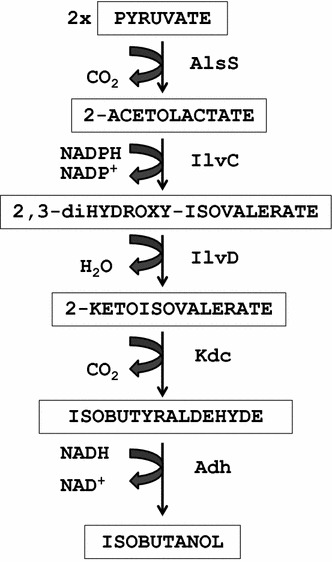
Pathway for isobutanol production. *AlsS* acetolactate synthase, *IlvC* acetohydroxy acid isomeroreductase, *IlvD* dihydroxy-acid dehydratase, *Kdc* 2-ketoacid decarboxylase, *Adh* alcohol dehydrogenase.

Atsumi et al. (2008, 2010) had designed a non-natural isobutanol production pathway consisting of five enzymatic steps from pyruvate (Figure 2). This pathway combines the *alsS, ilvC, kivD,* and *ADH2* genes that were independently cloned from *B. subtilis*, *E. coli*, *L. lactis* and *S. cerevisiae*, respectively and expressed in several transcription units harbored in at least two compatible plasmids (Atsumi et al. 2008, 2010). The use of two plasmids and different transcription units make this system complex to handle and thus, based on this work we have explored the possibility of designing a new single synthetic isobutanol operon (IbPSO) (see “Materials and methods”) to facilitate the cloning and manipulation of this non-natural pathway.

To improve the design of the gene arrangement and reduce its complexity, we have introduced three novelties; (i) the genes were de novo chemically synthesized using a programmed codon use optimal for different bacteria; (ii) the genes were cloned together creating a single transcription unit under the control of a single regulated promoter that can be harbored by a single broad-host-range plasmid or transposon; and (iii) the gene encoding the alcohol dehydrogenase (ADH2) responsible for the last biochemical step (the transformation of 2-isobutyraldehyde to isobutanol) has not been included in the IbPSO operon because as demonstrated by Atsumi et al. (2010) many bacteria possess Adh enzymes that can supply this activity and its inclusion in the operon does not provide additional advantages.

As a proof of concept we have constructed two broad-host range plasmids, pIZIbPSO and p424IbPSO, harbouring the IbPSO operon using *E. coli* DH10B (K12 strain) and *E. coli* CC118 (K12 strain) as hosts, respectively. Plasmid p424IbPSO derives from the synthetic pSEVA424 plasmid, and therefore the new construction has the novelty of being one of the few complete chemically synthetic plasmids used for metabolic engineering so far.

The plasmids were stably maintained in these strains and we confirmed that *E. coli* DH10B (pIZIbPSO) and *E. coli* CC118 (p424IbPSO) produced 0.9 and 1.4 g l^−1^ of isobutanol, respectively, using glucose as carbon source. These strains only produced isobutanol in the presence of IPTG confirming that the expression of the operon is under the control of the LacI regulator, as expected. These results demonstrated that the new IbPSO synthetic operon was fully functional in *E. coli*.

The plasmids extracted from these bacteria were further used to create other isobutanol producers with a single transformation event. Because both plasmids have two different broad-host range replication origins and two different antibiotic resistance markers they can be used to transform a large variety of hosts.

### Isobutanol overproduction in recombinant strains expressing IbPSO operon

To test the feasibility of producing isobutanol using the IbPSO operon different strains of *Escherichia*, *Pseudomonas, Shimwellia* and *Klebsiella* were transformed with plasmids pIZIbPSO or p424IbPSO depending of the strains and the possibility of using their antibiotic markers (data not shown). Remarkably, although we were able to obtain antibiotic resistant recombinants in all cases, the plasmids were partially deleted in several hosts. Obviously, only the transformants keeping intact plasmids were able to produce isobutanol in different amounts ranging from milligrams to grams per litter using glucose as carbon source. These results demonstrated that the stability of the plasmids and the isobutanol production were strongly dependent on the host strain. The reasons why the IbPSO containing plasmids were deleted in some strains or why some strains produced more isobutanol than others have not been investigated because the objective was to obtain a battery of isobutanol producers to select the best producing bacteria.

The two best isobutanol producers were *E. coli* W (ATCC9637) (pIZIbPSO) and *S. blattae* (p424IbPSO) that were selected for further analyses. The so called “Waksman’s strain” or “W strain”, *E. coli* W (ATCC9637) is a fast-growing industrial strain and the only safe (GRAS) strain that can grow using sucrose as sole carbon and energy source. In addition, this strain is able to produce high amounts of l-valine (Park et al. 2011). On the other hand, *S. blattae* is a non-pathogenic strain that has been used to produce high amounts of 1,3-propanediol (Heinrich et al. 2013).

**Figure 3:**
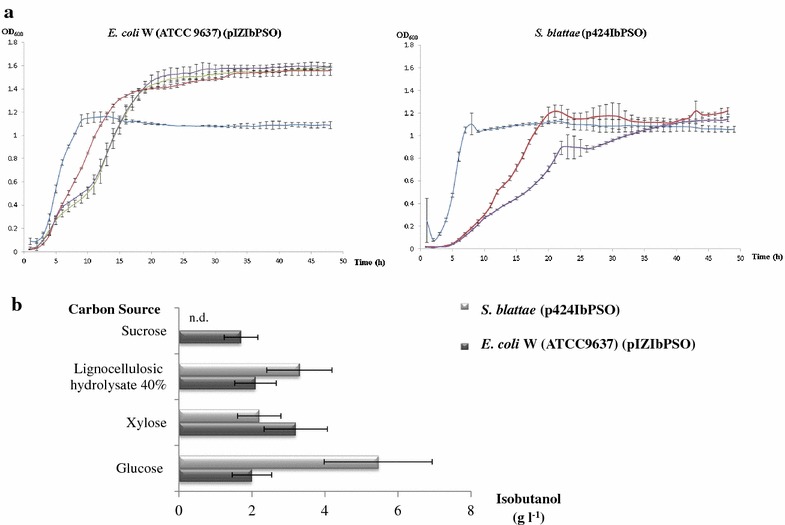
Growth curves and isobutanol production by *E. coli* W (ATCC 9637) (pIZIbPSO) and *S. blattae* (p424IbPSO). **a** Growth curves in different carbon sources. Glucose (20 g l^−1^) (*red*), xylose (20 g l^−1^) (*purple*), 20% lignocellulosic hydrolysate (20 ml liquid hydrolysate/100 ml medium) (*blue*) and sucrose (20 g l^−1^) (*green*). **b** Isobutanol production of the recombinant strains at 48 h in the presence of different carbon sources. *S. blattae* does not use sucrose as carbon source and the isobutanol production was not determined in this medium (nd).

Figure 3a shows the growth curves of the two strains in the different compounds used as carbon sources to measure the isobutanol production. The best isobutanol producer was *S. blattae* (p424IbPSO) (6 g l^−1^) when the microorganism was cultured on glucose, but isobutanol production was also high (>2 g l^−1^) when this strain was cultured in the presence of lignocellulosic hydrolysate or xylose. The strain *E. coli* W (ATCC 9637) (pIZIbPSO) produced up to 3 g l^−1^ in the presence of xylose and 2 g l^−1^ when glucose or lignocellulosic hydrolysate were metabolized, but more important this strain also produced 1.7 g l^−1^ of isobutanol with sucrose as carbon source. This is the first time that isobutanol has been produced from sucrose. The yields of the transformations are shown in Table 2.

**Table 2:** Isobutanol production yields (g/g) with the carbon sources tested

	*E. coli* W	*S. blattae*
Y_isobutanol/glucose_	0.095	0.196
Y_isobutanol/xylose_	0.160	0.123
Y_isobutanol/lignocellulosic hydrolysate_^a^	0.067	0.102
Y_isobutanol/sucrose_	0.204	n.d.

^a^24 g l^−1^ glucose and 12 g l^−1^ xylose.

The results demonstrate the potential of the IbPSO strategy, as significant isobutanol productions were achieved even without a comprehensive optimization of the strains and of the operation conditions.

### Testing strain tolerance to isobutanol and norvaline

To investigate whether the higher productions observed in *E. coli* W and *S. blattae* correlate with their tolerance to isobutanol and/or their overproduction of branched amino acids we tested their resistance to isobutanol and to norvaline, respectively.

**Figure 4:**
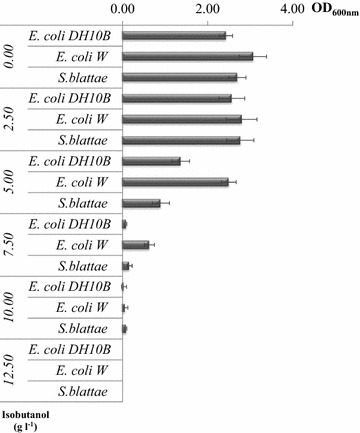
Isobutanol tolerance. Strains were cultured in liquid medium in the presence of increasing concentrations of isobutanol.

Isobutanol is known to be toxic to microbial cells (Brynildsen and Liao 2009) and this feature can be a major restriction to improve productivity. To check isobutanol tolerance the host strains were cultured in liquid medium in the presence of increasing concentration of isobutanol. Figure 4 shows that *E. coli* W (ATCC 9637) was tolerant to isobutanol up to 7.5 g l^−1^. However, *E. coli* DH10B (K12 strain) and *S. blattae* were less tolerant. None of the tested strains were tolerant to more than 10 g l^−1^ of isobutanol.

Taking into account that norvaline resistance is a property that is related with an improved ability for overproducing branched amino acids in bacteria and that was used for increasing the isobutanol production in *E. coli* (Smith and Liao 2011) we have observed that the better producers *E. coli* W (ATCC 9637) and *S. blattae* are able to grow up to 10 mg l^−1^ of norvaline in contrast with *E. coli* DH10B that only resist up to 3 mg l^−1^ of norvaline. This result confirmed the previous finding that *E. coli* W can grow in media containing up to 50 mM norvaline and 2 mM valine (Park et al. 2011). This result might also explain why *E. coli* DH10B (pIZIbPSO) in spite of being highly resistant to isobutanol (5 g l^−1)^ only produced 0.7 g l^−1^ of isobutanol from glucose as carbon source. Therefore, resistance to norvaline can be a selective trait to identify the best potential isobutanol producers.

## Discussion

The pioneering work of Liao and co-workers demonstrated that it was possible to transform glucose into isobutanol using engineered microorganisms (Atsumi et al. 2008; Atsumi and Liao 2008; Connor and Liao 2009; Gronenberg et al. 2013). To achieve this objective the producer organism must first convert the carbon source to central metabolic intermediates, which are then transformed into isobutanol through engineered pathways. When using non-conventional resources strain engineering efforts must address problems in both resource utilization and isobutanol synthesis. Therefore, it would be possible to construct the feedstock utilization pathways into an organism that already produces isobutanol, or construct the isobutanol production pathways into a host organism capable of metabolizing the desired resource. The choice of the strategies depends on the complexity of the two types of pathways and difficulties in expressing the enzymes involved (Atsumi et al. 2008; Atsumi and Liao 2008; Connor and Liao 2009; Gronenberg et al. 2013).

With the sole exception of plasmid pJL26 used to produced isobutanol in *Ralstonia eutropha* (Lu et al. 2012) the existing engineered isobutanol production pathways have been constructed using the native genes obtained from different organisms and these genes have been cloned in pairs or individually in different plasmids. Obviously, this strategy presents several problems: (i) the codon use of the genes were not adapted to the host organism; (ii) the genes are expressed in different plasmids which requires the use of several antibiotics and hampers their stability; (iii) the requirement of two or more plasmids complicates their transference to alternative hosts; and (iv) the genes are constructed in different operons making more difficult its regulation.

In order to circumvent these problems we have engineered a completely synthetic pathway in a single operon. The new tools of synthetic biology allowed us not only to optimize the codon usage of the genes but also their transcriptional and translational issues. The genes were placed in the operon according to their metabolic order in the pathway, as occurs on many natural operons. In this way, the complete operon can be uniformly controlled and inserted in a broad range plasmid that can be tested in many different bacteria having diverse metabolic properties.

As a proof of concept we have inserted the new IbPSO operon in two different broad-host range expression vectors, pIZ1016 and pSEVA424, under the control of *Ptac* and *Ptrc* promoters, respectively. Therefore, these plasmids allow testing isobutanol production in many different bacteria. Taking into account that pSEVA424 is also a synthetic plasmid, the construction p424IbPSO is a completely synthetic product.

It is worth to mention that based on the results of Atsumi et al. (2010) we have excluded from the operon the gene encoding for an alcohol dehydrogenase since they have demonstrated that *E. coli* and other bacteria already contain encoded in their chromosomes wide-substrate-range dehydrogenases (e.g., YqhD or AdhA in *E. coli*) that can efficiently perform the last enzymatic step, i.e., isobutyraldehyde reduction, even better than ADH2 from *S. cerevisiae* currently used in previous constructions. Moreover, the chromosome expression of these enzymes appears to saturate the enzyme requirements for isobutanol production (Atsumi et al. 2010).

The best isobutanol producer strains constructed so far must be equipped with different mutations in order to achieve a high isobutanol yield, because the recombinants based on the transformation of wild type strains accumulate other intermediates and produce very low isobutanol yields (Atsumi et al. 2008; Atsumi and Liao 2008; Connor and Liao 2009; Gronenberg et al. 2013). One of the best producer strains is *E. coli* JCL206, a multiple mutant strain (*ΔadhE*, *ΔfrdBC*, *Δfnr*, *ΔldhA*, *Δpta*, *ΔpflB*) derived from *E. coli* K12 strain BW25113, bearing two plasmids, pSA65 and pSA69, that produces about 7 g l^−1^ of isobutanol in flasks (Atsumi et al. 2008, 2010) when cultured in similar conditions to that used in this work. This suggests that the recombinant *E. coli* W (ATCC 9637) and *S. blattae* strains are good candidates to be improved as isobutanol producers. In this sense it is important to mention that *E. coli* JCL206 (pSA65, pSA69) can produce up to 22 g l^−1^ when cultured in higher concentrations of glucose and longer times (Atsumi et al. 2008) or even higher (about 50 g l^−1^) in bioreactors with in situ product removal (Baez et al. 2011). In this sense, it is very difficult to compare the isobutanol productions described in the literature because when the recombinant bacteria are cultured in flasks, the alcohol is partially evaporated during the fermentation process. Then, to reduce the isobutanol evaporation, flasks must be sealed causing microaerophilic fermentation conditions that do not favour the bacterial growth and are difficult to control and reproduce.

Whether, our recombinant strains of *E. coli* W (ATCC 9637) and *S. blattae* can be improved to reach the high isobutanol productions using similar operation conditions that those described for the best producer strains is under study. Moreover, the construction of specific mutants of these new hosts to reduce by-products might still open the possibility of obtaining even best producer strains as demonstrated for other microorganisms (Atsumi et al. 2008; Atsumi and Liao 2008; Connor and Liao 2009; Gronenberg et al. 2013). Nevertheless, it is interesting to notice that during isobutanol productions we have not observed in our recombinant strains a significant accumulation in the culture medium of metabolites such as lactic and acetic acids that might reduce the production of isobutanol (data not shown).

On the other hand, the reasons why *E. coli* W (ATCC 9637) and *S. blattae* are more robust, produce more isobutanol, and support stably these plasmids better than others tested strains, remain unknown. The analysis of their central and amino acid metabolism based on their genome information did not provide any specific evidence of the existence of specific pathways that might contribute to their robustness. Nevertheless, we suggest that the finding that they are naturally resistant to high concentrations of norvaline might contribute positively.

To produce isobutanol cost effectively on an industrial scale, more economical carbon, nitrogen, and mineral sources should be tested to replace glucose. Sucrose is one of the main feedstock for industrial fermentations presenting several advantages over glucose, such as the fewer amounts of energy, water, and chemicals used for its production which lies in its lower price. Waste molasses from beet and sugar cane contain large amounts of sucrose and they have been used at industrial level as raw materials for different fermentation processes. However, most industrial *E. coli* strains are derived from the wild types that do not utilize sucrose. In this sense *E. coli* W (ATCC 9637) is able to metabolize sucrose by using the chromosomally encoded sucrose catabolism (*csc*) regulon. In this work we have demonstrated for the first time that *E. coli* W (ATCC 9637) (pIZIbPSO) can produce isobutanol from this important raw material. This robust strain appears to be an excellent platform for industrial uses due to its more rapid growth with much less by-product formation compared with *E. coli* K-12 strains as suggested by Park et al. (2011). On the other hand, the observation that *S. blattae* and *E. coli* W (ATCC 9637) grow well on the highly toxic crude lignocellulosic hydrolysates (Figure 3a), has allowed us to produce isobutanol directly using these slurries as carbon sources even containing such toxic mixtures. Because these strains can consume glucose, xylose and arabinose that are the main sugar components of these hydrolysates, these bacteria appear to be ideal for developing an isobutanol production process based on the fermentation of such slurries.

Summarizing, the proof of concept provided in this work opens new avenues to investigate the production of isobutanol using the tools of synthetic biology in order to optimize the production pathways in a simple way and to investigate the utility and adaptability of alternative host strains having different metabolic capabilities to use alternative raw materials or able to work under extreme toxic conditions.
